# Current radiotherapy for recurrent head and neck cancer in the modern era: a state-of-the-art review

**DOI:** 10.1186/s12967-022-03774-0

**Published:** 2022-12-06

**Authors:** Yue Li, Yuliang Jiang, Bin Qiu, Haitao Sun, Junjie Wang

**Affiliations:** Department of Radiation Oncology, Peking University Third Hospital, 49 North Garden Road, Haidian District, Beijing, 100191 China

**Keywords:** Head and neck cancer, Recurrence, External beam radiotherapy, Brachytherapy

## Abstract

**Background:**

In the management of head and neck cancer (HNC) patients, local recurrence is a common cause of treatment failure. Only a few patients with recurrent HNC (rHNC) are eligible for salvage surgery and the majority of patients receive systemic therapy and radiotherapy. In recent years, with the development of irradiation technology, radiotherapy for rHNC patients has markedly attracted clinicians’ attention and its therapeutic effects on patients with end-stage cancer are worthy of investigation as well.

**Methods:**

Several studies have investigated the role of radiotherapy in the treatment of rHNC patients. We reviewed retrospective reports and prospective trials published in recent decades that concentrated on the management of rHNC.

**Results:**

A growing body of evidence supported the application of irradiation to rHNC patients. According to the results of this review, current radiotherapy could achieve a better efficacy with a lower incidence of toxicity.

**Conclusion:**

Radiotherapy is a promising treatment for rHNC patients.

## Introduction

Head and neck cancer (HNC) is a broad term, including epithelial malignancies that occur in the paranasal sinuses, nasal cavity, oral cavity, pharynx, and larynx. Almost all these malignancies are head and neck squamous cell carcinoma (HNSCC). Approximately two-thirds of HNSCC patients have the advanced-stage disease with regional lymph nodes. The initial appearance of distant metastasis is uncommon only affecting approximately 10% of the patients [1]. HNC is the seventh most common cancer in the world, which is typically diagnosed in elderly patients associated with large amounts of tobacco and alcohol use. Besides, cases of human papillomavirus (HPV)-associated oropharyngeal cancer, which is mainly caused by HPV-16, are increasing in recent years, and this type of HNC is associated with a better prognosis than HPV-negative oropharyngeal cancer [2].

Despite the advancement of modern HNC treatment modalities, cancer recurrence is still a major problem, with a locoregional recurrence rate of 15–50% [3]. Of the HNSCC asymptomatic recurrences after definitive radiotherapy and chemotherapy, 93% are local or regional, and they mainly occur within the first 2 years after the initial treatment [4, 5]. Salvage surgery may be a curative option for patients with resectable locoregional recurrence [1]. For recurrent HNC (rHNC), only 15–30% of patients are indicative for surgery and the 5-year survival rate is 16–36% [6]. In the majority of cases, salvage surgery is not feasible, or it is only possible with severe complications and limited success rates [7]. When surgical treatment is not possible, the prognosis of patients with recurrent HNSCC (rHNSCC) is unsatisfactory. The median survival of untreated patients, receiving palliative chemotherapy, and conventional radiotherapy is 3–5, 6–9, and 9–14 months, respectively [8].

In the past, palliative chemotherapy was the main choice for patients with inoperable rHNC who had received high-dose radiotherapy. With the emergence of new technologies, the re-irradiation of recurrent tumors has markedly attracted clinicians’ attention [9]. Considering the high incidence of re-irradiation toxicities, for rHNC, especially in cases that have previously undergone radiotherapy, it is highly necessary to adopt a radiotherapy program with a high conformability and an accurate dose distribution, to reduce adverse reactions to normal tissues.

In recent years, several studies have shown that current radiotherapy technologies can effectively treat rHNC and result in a better prognosis for rHNC patients. These studies assessed the survival and local tumor control of patients, and adopt Common Terminology Criteria for Adverse Events (CTCAE), Radiation Therapy Oncology Group (RTOG) morbidity scoring criteria, or European Organization for Research and Treatment of Cancer (EORTC) Late Radiation Morbidity Score to evaluate adverse effects. In the present review, the efficacy and safety of intensity-modulated radiation therapy (IMRT), stereotactic body radiation therapy (SBRT), high-dose-rate brachytherapy (HDR-BRT), and low-dose-rate brachytherapy (LDR-BRT) in the treatment of rHNC patients were investigated.

## IMRT

Although the role of re-irradiation is controversial due to concerns about the high incidence of severe chronic toxicity, it is still a potentially curative treatment option for patients with locoregionally recurrent tumors. The clinical application of IMRT can provide effective biological doses for more conformal areas to improve tumor control while minimizing treatment-related toxicities [10–12].

Lee et al. reviewed the efficacy of re-irradiation using IMRT for recurrent or second primary HNC (RSPHNC). From 2007 to 2018, a total of 17 studies were included in this review, involving 1635 patients. Except for a study with a median dose of 49 Gy, the re-irradiation dose ranged from 59.4 to 70 Gy, which did not significantly vary among different studies. The 2-year local control (LC) and overall survival (OS) rates were 52% (95% confidence interval [CI], 46%-57%) and 46% (95% CI, 41%-50%) with re-irradiation using IMRT, respectively. The pooled rates of late grade ≥ 3 and grade 5 toxicities were 26% (95% CI, 20%-32%) and 3.1% (95% CI, 2%-5%), respectively. In the subgroup analysis, the salvage surgery rate (< 42% vs. ≥ 42%) affected the 2-year LC rate (45.9% vs. 58.5%, P = 0.011) [13]. Another retrospective cohort study conducted in 2020 showed primary subsite (non-larynx/hypopharynx/oral cavity), recurrent tumor size (< 3 cm), the interval between radiotherapy (RT) courses (≥ 24 months), and salvage surgery were found to be associated with a longer OS, while the interval between RT courses (≥ 24 months) and salvage surgery were noted as positive prognostic factors of LC. The authors suggested that a longer interval from the previous RT course was associated with better LC and less aggressive recurrent disease. However, this study did not determine the prognostic impact of HPV on patients, as HPV status was only available in few patients with oropharyngeal cancer [14]. Caudell et al. conducted a multi-institution retrospective cohort study to investigate the effects of selective treatment volume, dose, and fraction on outcomes and toxicity. Their results indicated that dose ≥ 66 Gy may be correlated with the improved prognosis of patients undergoing definitive re-IMRT. Postoperatively, after treating gross disease, the dose of 50–66 Gy was found sufficient. Although the 2-year OS was 60% for patients with HPV+ recurrence or second primary oropharyngeal cancer compared with 39.5% for patients with HPV-negative cancer, this result did not reach statistical significance. Moreover, hyperfractionation and elective neck irradiation had no significant benefits, and they may increase toxicity [15].

In conclusion, IMRT is advantageous for the treatment of rHNC patients, especially because of the high conformity of the target volume and the optimized sparing of previously irradiated organs at risk (OARs) [16].

## SBRT

SBRT is a form of modern conformal high-precision external beam radiotherapy (EBRT) that can provide various radiobiological benefits in diverse clinical conditions. SBRT can deliver dose in multiple small beams (> 150 per treatment) with less skin toxicity and a smaller irradiated volume, and it may allow the implementation of a hypofractionated scheme [17]. The SBRT aims to provide highly precise and ablative doses of radiation for the radiotherapy of the target area. For the therapy of recurrent HNSCC, SBRT can increase the dose to 50 Gy in 5 divided doses [3]. Therefore, SBRT possesses the advantages of shorter treatment duration and avoids interruption of systemic treatment. SBRT can generate a steep dose gradient between target tissues and surrounding healthy tissues, thereby reducing the overall radiation dose to critical organs at proximity to the target volume [18, 19]. The results of recent studies on the treatment of rHNC with SBRT are summarized in Table 1 [3, 8, 9, 17, 20–23].

**Table 1:** Results of irradiation using SBRT

Study	Study design	No. of patients	Previous radiotherapy	Follow-up, mo	Tumor volume, median (range), cm^3^	No. of fractions, median (range)	Total dose, median (range), Gy	Efficacy	Severe toxicity	References
Jean-Claude Rwigema, 2010	R	85	Median time to previous irradiation: 19.4 mo (range, 1.0–601.8 mo); Median prior BED10: 70 Gy (range, 32–170.7 Gy)	6 (1.3–39)	Tumor volume: 25.1 (2.5–162)	5 (1–5)	35 (15–44)	1-year LC: 51.2%; 2-year LC: 30.7%; 1-year OS: 48.5%; 2-year OS: 16.1%; Median OS: 11.5 mo	Grade 3 acute toxicities: 4.7%	[8]
Jean-Claude Rwigema, 2011	R	96	Median interval to failure from prior irradiation: 16 mo (range, 4.6–423.1 mo); Median dose of prior irradiation: 68.4 Gy (range, 32–170.7 Gy)	14 (2–39)	GTV: 24.3 (2.5–162)	5 (1–5)	35 (15–50)	1-year OS: 58.9%; 2-year OS: 28.4%; Doses 40 to 50 Gy: 1-year LRC: 69.4%; 2-year LRC: 57.8%; 3-year LRC: 41.1%; Doses 15 to 36 Gy: 1-year LRC: 51.9%; 2-year LRC: 31.7%; 3-year LRC: 15.9%	Grade 3 acute toxicities: 5.2%; Grade 3 late toxicities: 3.1%	[3]
Mustafa Cengiz, 2011	R	46	Median time to previous irradiation: 38 mo (range, 3.8–306 mo); Median dose of prior irradiation: 61 Gy (range, 30–70 Gy)	7 (2–23)	Tumor volume: 45 (3–206)	5 (1–5)	30 (18–35)	1-year PFS: 41%; Median PFS: 10.5 mo; 1-year OS: 46%; Median OS: 11.93 mo	Grade 3 acute dermatitis and mucositis: 4.4%; Grade ≥ 2 late toxicities: 13.3%	[9]
Bénédicte Comet, 2012	P	40	Median time to previous irradiation: 31.6 mo (range, 7.9–263.7 mo); Median dose of prior irradiation: 66 Gy (range, 18–192 Gy)	25.6	PTV: 64.1 (4.7–295.6)	6	36	Median relapse-free survival: 8.8 mo; Response rate: 79.4%; 1-year OS: 58%; 2-year OS: 24%; Median OS: 13.6 mo	Grade 3 toxicities: 10.3%	[17]
Pierluigi Bonomo, 2014	R	17	Median time to previous irradiation: 24 mo (range, 10–168 mo); Median dose of prior irradiation: 66 Gy (range, 50–70 Gy)	7.5 (2–17)	GTV: 58.7 (8.5–211.3)	5	30 (25–35)	CR: 25%; PR: 31%; SD: 44%	Grade 3 mucositis: 6%	[20]
Hideya Yamazaki, 2016	R	107	Median time to previous irradiation: 14.5 mo (range, 0.7–1180 mo); Median dose of prior irradiation: 60 Gy (range, 40–116 Gy)	15 (10–122)	PTV: 28.4 (1–339)	5 (3–8)	30 (15–39)	2-year LRC: 64%; 1-year OS: 55%; 2-year OS: 35%; Median OS: 14.4 mo	Grade ≥ 3 toxicities: 21%	[21]
John A Vargo, 2018	Retrospective comparative analysis	197	Median time to previous irradiation: 14.4 mo (range, 1.2–420 mo); Median dose of prior irradiation: 70 Gy (range, 40–170.7 Gy)	7.1 (1–120)	GTV: 30 (1–427)	5 (1–8)	40 (16–50)	Cumulative LRF: 57.0%; 2-year OS: 16.3%; Median OS: 7.8 mo	Grade ≥ 3 acute toxicities: 11.7%; Grade ≥ 3 late toxicities: 11.6%	[22]
Luke Stanisce, 2018	R	25	Median time to previous irradiation: 14 mo (range, 3–72 mo); Median dose of prior irradiation: 70 Gy (range, 30–110 Gy)	NA	GTV: 31.75 (5.5–121.8)	5 (3–5)	40 (24–44)	LF: 32%; Median LF: 5.8 mo; LRF: 28%; Median LRF: 8.5 mo; 1-year OS: 32%; 2-year OS: 16%; Median OS: 7.5 mo	Grade 3 acute toxicities: 4%; Grade 3 late toxicities: 6%	[23]

*R* retrospective analysis, *P* prospective clinical trial, *mo* months, *NA* not available, *BED* biologically effective dose, *GTV* gross tumor volume, *PTV* planning target volume, *LC* local control, *OS* overall survival, *LRC* locoregional control, *PFS* progression-free survival, *LRF* locoregional failure, *LF* local failure, *CR* complete response, *PR* partial response, *SD* stable disease

In recent years, numerous studies have evaluated the safety and outcomes of SBRT in patients with HNC who had previously received radiotherapy. Rwigema et al. enrolled 85 rHNSCC patients (23 patients with distant metastasis at the time of treatment) who received SBRT at a dose of 15–44 (mean, 35) Gy, and 33% of patients received cetuximab concurrently. The 1- and 2-year LC and OS rates were 51.2% and 30.7%, and 48.5% and 16.1%, respectively. The median survival was 11.5 (range, 3–51) months. For patients without distant metastasis, the 1- and 2-year OS rates were 61.9% and 23.4%, respectively. The treatment was well-tolerated, and there were no grade 4 or 5 treatment-related toxicities. Moreover, within 6 months of the median follow-up, those patients who received SBRT < 35 Gy had significantly lower LC than those with ≥ 35 Gy, with a similar incidence rate of toxicity [8].

Rwigema et al. evaluated 96 patients with unresectable, previously irradiated rHNSCC, who received SBRT at the relapse sites, and 39 patients (40.6%) who received cetuximab concurrently in a retrospective cohort study. The 1-, 2-, and 3-year locoregional control (LRC) rates for a dose of 40–50 Gy were 69.4%, 57.8%, and 41.1%, respectively, and those of 15–36 Gy were 51.9%, 31.7%, and 15.9%, respectively. The incidence rates of grade 1, 2, and 3 acute toxicities were 37.5%, 17.7%, and 5.2%, respectively, while those of grade 1, 2, and 3 long-term complications were 16.7%, 9.3%, and 3.1%, respectively. This study demonstrated that it is feasible to increase the SBRT dose to 50 Gy in 5 fractions. Moreover, a higher SBRT dose was associated with a significantly higher LRC rate. Compared with a small gross tumor volume (GTV ≤ 25 cm^3^), a large tumor volume (GTV > 25 cm^3^) requires a higher SBRT dose to achieve the best response rate [3].

Cengiz et al. enrolled 46 rHNC patients, and 30 patients of them were histopathologically diagnosed with squamous cell carcinoma. The median dose of SBRT was 30 Gy (range, 18–35 Gy) in 5 fractions. Of the 37 patients who were evaluated for the treatment response, 10 (27%), 11 (29.8%), and 10 (27%) patients achieved complete response (CR), partial response (PR), and stable disease (SD), respectively. Besides, 31 (83.8%) patients achieved local disease control. The median OS was 11.93 months, and the median progression-free survival (PFS) was 10.5 months. The 1-year PFS and OS rates were 41% and 46%, respectively. Long-term complications of grade 2 or greater were observed in 6 (13.3%) patients. During the follow-up, 8 (17.3%) patients developed carotid blow-out syndrome, and 7 (15.2%) patients died of carotid artery hemorrhage. This fatal syndrome only occurs in patients who had tumors around the carotid artery and received all the prescribed doses [9].

Comet et al. prospectively enrolled 40 patients who had inoperable recurrent, or new primary HNC in previously irradiated areas. All patients received SBRT at a dose of 36 Gy in 6 fractions, 15 patients received concomitant cetuximab, and 1 patient received concomitant cisplatin. The median follow-up was 25.6 months, of whom 34 patients could be assessed for tumor response. The median OS was 13.6 months, and the response rate was 79.4% (15 and 12 patients achieved CR and PR, respectively). In addition, grade 3 toxicity occurred in 4 patients [17].

Yamazaki et al. assessed the prognosis of 107 patients with rHNC after re-irradiation with SBRT using a CyberKnife. The 2-year OS rate was 35%. Important prognostic factors for a longer OS were the primary site (nasopharynx), absence of ulceration, and the planning target volume (PTV) ≤ 40 cm^3^. This study recorded 22 serious toxicities, including 11 patients with carotid blow-out syndrome (CBOS), 9 of whom died. Since CBOS was the only fatal toxicity found after re-irradiation, ulceration affected OS through CBOS. In addition, unlike the alternate-day treatment schedules of other studies, this study used daily treatment, which may have increased toxicity [21].

Stanisce et al. evaluated the relevant outcomes of stereotactic body radiotherapy treatment for recurrent, previously irradiated HNC. This study enrolled 25 patients who were treated with CyberKnife. In total, 11 patients (44%) received concurrent cetuximab chemotherapy during re-irradiation. The median survival of all patients was 7.5 (range, 1.5–47.0) months, and the median survival of 20 (80%) patients who received curative treatment was 8.3 months. Besides, 1-year survival of the entire population was 32%. The 1- and 2-year survival rates of the curative sub-cohort were 40% and 20%, respectively. There were 8 (32%) and 7 (28%) patients with local and locoregional failure, respectively. The incidence rate of grade 3 acute toxicity was 4%, while that of grade 3 late toxicity was 6% [23].

To compare the efficacy of SBRT and IMRT, Vargo et al. enrolled 414 unresectable recurrent or second primary squamous cell carcinoma of the head and neck patients, of whom 217 received IMRT and 197 received SBRT. Of the IMRT patients, 84% received systemic therapy. The unadjusted 2-year OS rate for IMRT and SBRT groups was 35.4% and 16.3%, respectively (P < 0.01). For recursive partitioning analysis (RPA) class III patients, the OS in IMRT and SBRT groups was similar. In all class II patients, IMRT was associated with an improved OS (P < 0.001). Further subgroup analysis showed that when small tumors received SBRT with a dose of ≥ 35 Gy, the OS was comparable. For large tumors, treatment with IMRT was associated with an improved OS (rT0-rT2 tumors were characterized as “small” tumors, and rT3-rT4 tumors were characterized as “large” tumors). The incidence rate of acute grade ≥ 4 toxicity in the IMRT group was greater than that in the SBRT group, and there was no significant difference in the incidence rate of late toxicity. In addition to the RPA class, younger age, second primary tumor, and small tumor volume were independently associated with the improvement of OS in the SBRT group [22].

In general, SBRT can be used to treat patients with rHNC, especially those who have previously received radiotherapy, and is considered inappropriate for re-irradiation by conventional methods. The 2-year LRC rate was 31.7–64%, the 1- and 2-year OS rate were 32–58.9% and 16–35%, and the median OS was 7.5–14.4 months for rHNC patients who were treated with SBRT. Moreover, higher SBRT dose was associated with better LRC, and younger age, the primary site (nasopharynx), absence of ulceration, second primary tumor, and small tumor volume were prognostic factors for OS. In the majority of patients, SBRT effectively alleviated the disease with less toxicity.

## HDR-BRT

In HNC, the conventional median survival of the patients receiving platinum (Pt)-based chemotherapy is about 6 months, and the recurrent rate after radical treatment can be as high as 30–50% [24]. In addition, recurrence mainly (80%) occurs in volumes that were previously exposed to high doses [25]. Therefore, due to the risks of toxicity, extreme morbidity, and mortality, re-irradiation with external beam is not possible in many cases. In addition, after previous treatment, less-defined anatomical location can hinder radiation [7]. Hence, the therapeutic advantages of brachytherapy for rHNC should be highlighted. Compared with EBRT, brachytherapy can deliver a high total dose directly to the tumor, and the rapid dose fall-off above PTV can protect surrounding normal tissues [6].

Brachytherapy is a type of radiotherapy, in which radionuclide sources are used to deliver radiation doses at a distance of up to a few centimeters by surface, intracavitary, intraluminal, or interstitial application. Brachytherapy, alone or in combination with EBRT, plays an important role in the treatment of diverse types of cancer. HDR-BRT uses radionuclides, such as Iridium-192, to irradiate a designated target point or volume at dose rates of 20 cGy per min (12 Gy per hour) or more. HDR-BRT is appropriate for the treatment of malignant or benign tumors, where the treatment volume or target point is defined and accessible [26]. HDR-BRT uses a remote after-loading source (most commonly Iridium-192) to deliver the dose through previously placed catheters or applicators. Decades of experience in HDR-BRT confirm its efficacy and safety [27]. Recent studies on the treatment of rHNC with HDR-BRT are summarized in Table 2 [6, 7, 24, 28–31].

**Table 2:** Results of irradiation using HDR-BRT

Study	Study design	No. of patients	Previous radiotherapy	Follow-up, mo	Tumor volume, median (range), cm^3^	No. of fractions, median (range)	Total dose, median (range), Gy	Efficacy	Severe toxicity	References
David J Perry, 2010	R	34	Median time to previous irradiation: 16 mo (range, 1–337 mo); Median dose of prior irradiation: 63 Gy (range, 24–74 Gy)	23 (6–54)	NA	1	15 (10–20)	1-year LPFS: 66%; 2-year LPFS: 56%; 1-year OS: 73%; 2-year OS: 55%; Median OS: 24 mo	Cellulitis: 14%; Fistula or wound complications: 9%; Osteoradionecrosis: 3%; Radiation-induced trigeminal neuralgia: 3%	[30]
V Rudzianskas, 2012	R	30	Median interval to failure from prior irradiation: 12 mo (range, 3–43 mo); Median dose of prior irradiation: 66 Gy (range, 50–72 Gy)	16 (4–32)	PTV: 36 (8–107)	12, bid	30	1-year LC: 73%; 2-year LC: 67%; 1-year DFS: 60%; 2-year DFS: 53%; 1-year OS: 63%; 2-year OS: 47%	Acute toxicities: Grade 2 moderate fibrosis: 7%; Grade 3 delayed wound healing: 3%; Late toxicities: Grade 2 dysphagia: 3%; Grade 2 persistent hoarseness: 3%; Grade 4 osteonecrosis: 3%	[28]
L Matthew Scala, 2013	R	76	Median time to previous irradiation: 24 mo (range, 3–240 mo); Median dose of prior irradiation: NA (46.8–80 Gy)	11	Median IORT field size: 5 cm × 6 cm (range: 1 cm × 2 cm to 11 cm × 17 cm)	1	12 (7.5–17.5)	1-year in-field control rate: 66%; 2-year in-field control rate: 62%; Median DFS: 12 mo; 1-year OS: 64%; 2-year OS: 42%; Median OS: 19 mo	Flaps revision: 4%; Nonfatal carotid hemorrhage: 1%; Vagal neuropathy: 1%	[31]
Susanne Wiegand, 2013	R	12	Median time to previous irradiation: 96 mo (range, 12 -180 mo); Median dose of prior irradiation: NA	NA	NA	NA (2–3 Gy per fraction), bid	(20–33)	Median OS: 8.5 mo	None	[7]
Viktoras Rudžianskas, 2014	RCT	32	Median time to previous irradiation: 14.9 mo (range, 3–26.1 mo); Median dose of prior irradiation: 66 Gy (range, 50–70 Gy)	NA	PTV: 34.8 (8–107)	12, bid	30	1-year LC: 77%; 2-year LC: 63%; Median LC: 28.1 mo; 1-year OS: 74%; 2-year OS: 67%; Median OS: 33.4 mo	Severe acute toxicities: 34.4%; Severe late toxicities: 3.1%	[6]
Luca Tagliaferri, 2015	R	9	Median interval to failure from prior irradiation: 28 mo (range, 11–60 mo); Dose of prior irradiation > 65 Gy	21	NA	12, bid	30	Median DFS: 12 mo; Median OS: 23 mo	Grade 3 acute toxicities: 11.1%; Grade 2 late toxicities: 11.1%	[24]
John V Hegde, 2018	R	20	Median interval to failure from prior irradiation: 9.1 mo (range, 2.4–351.6 mo); Median dose of prior irradiation: NA	11.3 (3.4–65.6)	CTV: 43.7 (7.6–155.6)	8 (2–12), bid	36.5 (16.25–48)	1-year LC: 55%; 1-year regional control: 62%; 1-year LRC: 38%; 1-year DMFS: 94%; 1-year OS: 77%	Acute grade 3 toxicities: Dysphagia: 25%; Soft tissue necrosis: 5%; Grade 3 to 4 late toxicities: 33%	[29]

*R* retrospective analysis, *RCT* randomized controlled trial, *mo* months, *NA* not available, *bid* twice daily, *IORT* intraoperative radiotherapy, *PTV* planning target volume, *CTV* clinical target volume, *LPFS* local progression-free survival, *OS* overall survival, *LC* local control, *DFS* disease-free survival, *LRC* locoregional control, *DMFS* distant metastasis-free survival

### Studies reporting the application of HDR-BRT

Rudzianskas et al. evaluated the results of hypofractionated accelerated computed tomography (CT)-guided interstitial HDR-BRT for 30 patients with previously irradiated rHNC (primary tumor site without nasopharynx), including 13 patients who underwent surgical resection, followed by HDR-BRT, as well as 17 patients who received solely HDR-BRT. All patients received 2.5 Gy twice a day for a total dose of 30 Gy. The 1- and 2-year OS rates of the whole cohort were 63% and 47%, respectively, while LC rates were 73% and 67%, respectively, and the disease-free survival (DFS) rates were 60% and 53%, respectively. Besides, 3% of patients experienced grade 3 and 4 late complications. The median OS of patients with tumor volume ≤ 36 cm^3^ was 22 months, and that of patients with tumor volume > 36 cm^3^ was 9.2 months. Moreover, the 2-year LC and 2-year OS were improved in patients who underwent surgical resection and HDR-BRT compared with cases who only received HDR-BRT [28].

Rudžianskas et al. compared the efficacy and toxicity of three-dimensional conformal radiotherapy (3D-CRT) and HDR-BRT in the treatment of rHNC (primary tumor site without nasopharynx). After randomization, 31 patients received 3D-CRT (50 Gy/25 fractions), of whom 48.4% had undergone surgery. There were 32 patients who received HDR-BRT (30 Gy/12 fractions), while 50% of them had undergone surgery. Furthermore, the 1- and 2-year OS rates in the HDR-BRT group were 74% and 67%, respectively, while those in the 3D-CRT group were 51% and 32%, respectively (P = 0.002). The 1-year and 2-year LC rates in the HDR-BRT group were 77% and 63%, respectively, compared with 47% and 25% in the 3D-CRT group, respectively (P < 0.001). In the HDR-BRT group, severe (grade 3 and 4) acute toxicities occurred in 11 (34.4%) patients, and those in the 3D-CRT group were recorded in 17 (54.8%) patients. For severe late toxicity, 11 (35.5%) patients in the 3D-CRT group and 1 (3.1%) patient in the HDR-BRT group were identified (P = 0.001)[6].

Hegde et al. reported the use of HDR-BRT in re-irradiation of 20 patients with rHNC (primary tumor site without nasopharynx, including cutaneous skin) or a new primary lesion within a previously irradiated field. These patients received different treatment plans to achieve curative or palliative intent, including definitive brachytherapy alone (1 patient), salvage surgery with adjuvant brachytherapy (5 patients), external beam (chemo) radiotherapy and brachytherapy boost (3 patients), palliative sequential chemotherapy with brachytherapy (2 patients), and palliative brachytherapy alone (9 patients). The 1-year LC and OS rates were 55% and 77%, respectively. For curative treatment in 11 patients, the 2-year LC and OS rates were 73% and 56%, respectively. For palliative intent in 9 patients, the 6-month LC rate was 65%. Besides, 33% of patients had grade 3 to 4 late toxicity. Furthermore, age > 70 years old was associated with a poor OS, while previous salvage surgery showed a trend to improve LC and OS [29].

In conclusion, based on the current research, the use of HDR-BRT only or the combination of debulking surgery and perioperative HDR-BRT was feasible in the treatment of patients with rHNC. The 1- and 2-year LC rate were 55–77% and 63–67%, the 1- and 2-year OS rate were 63–77% and 47–67%, and the median OS was 8.5–33.4 months. Additionally, surgical resection was associated with better LC, and small tumor volume, surgical resection, younger age would improve OS.

### Studies reporting the application of HDR intraoperative radiotherapy (IORT)

IORT is an important approach to improve the prognosis of HNC patients undergoing definitive surgery. HDR-IORT is defined as single high-dose radiation when the tumor bed is exposed during surgery, which can be delivered with photons from a high-dose-rate gamma-emitting radioisotope, including Iridium 192 (Ir-192 HDR-IORT). Moreover, the combination of HDR-IORT and EBRT results in several advantages: (1) while accurately defining the tumor bed, it provides conformal high-dose radiation; (2) potential reduction of the dose of subsequent EBRT; (3) shortening the overall treatment time; (4) increase of the dose [32].

Perry et al. reported the use of HDR-IORT for the treatment of rHNC. In total, 34 rHNC patients with prior EBRT received a single fraction (10–20 Gy) of HDR-IORT after complete surgical resection of the recurrent disease. The IORT was delivered using an afterloader device with an Iridium-192 source. Subsequently, 5 patients received EBRT as a consolidation treatment (median dose, 50 Gy; range, 30–63 Gy), and 7 patients received chemotherapy. The 1- and 2-year local progression-free survival (LPFS) rates were 66% and 56%, respectively, and 13 cases (34%) had in-field recurrence. The 1- and 2-year distant metastasis-free survival rates were 81% and 62%, respectively. The 1- and 2-year OS rates were 73% and 55%, respectively, and the median OS was 24 months. For patients undergoing salvage surgery for previously irradiated rHNC, IORT could improve LC with an acceptable toxicity rate. The application of this method has failed to improve OS, highlighting the need for improved systemic treatment; however, the role of LC in patients’ quality of life should not be underestimated [30].

Scala et al. introduced the application of HDR-IORT to treat 76 patients with rHNC (primary tumor site without nasopharynx), and 24% of patients received postoperative EBRT to a median dose of 45 Gy. The 2-year estimate of in-field tumor control was 62%, and the median OS of all patients was 19 months. It was found that the survival of patients who achieved in-field control was significantly prolonged compared with those of in-field progression (33 vs. 17 months, P = 0.01)[31].

In patients who received salvage EBRT, locoregional control proved to be critical for improving OS. For postoperative HDR, interstitial brachytherapy, which was defined as the use of catheters that are placed in and around a tumor for several days, could increase the risk of infection and the length of hospitalization; on the contrary, IORT was completed intraoperatively, and it could retract and protect surrounding normal tissues [30]. In summary, as a treatment that combines surgery and HDR, HDR-IORT allows the irradiation dose to be applied to areas that are more likely for harboring disease, while preserving the deeper structures that have been exposed during the prior EBRT courses. However, the therapeutic effects of HDR-IORT remain to be further investigated.

## LDR-BRT

Brachytherapy refers to the use of radionuclides to treat malignant tumors or benign diseases through radioactive sources placed close to or into tumor or treatment site [33]. LDR-BRT is accomplished through permanent implants, in which the radioactive sources are permanently placed into cancerous tissues. At the designated point, LDR-BRT is delivered at a dose rate of 4–200 cGy per hour [33].

**Fig. 1:**
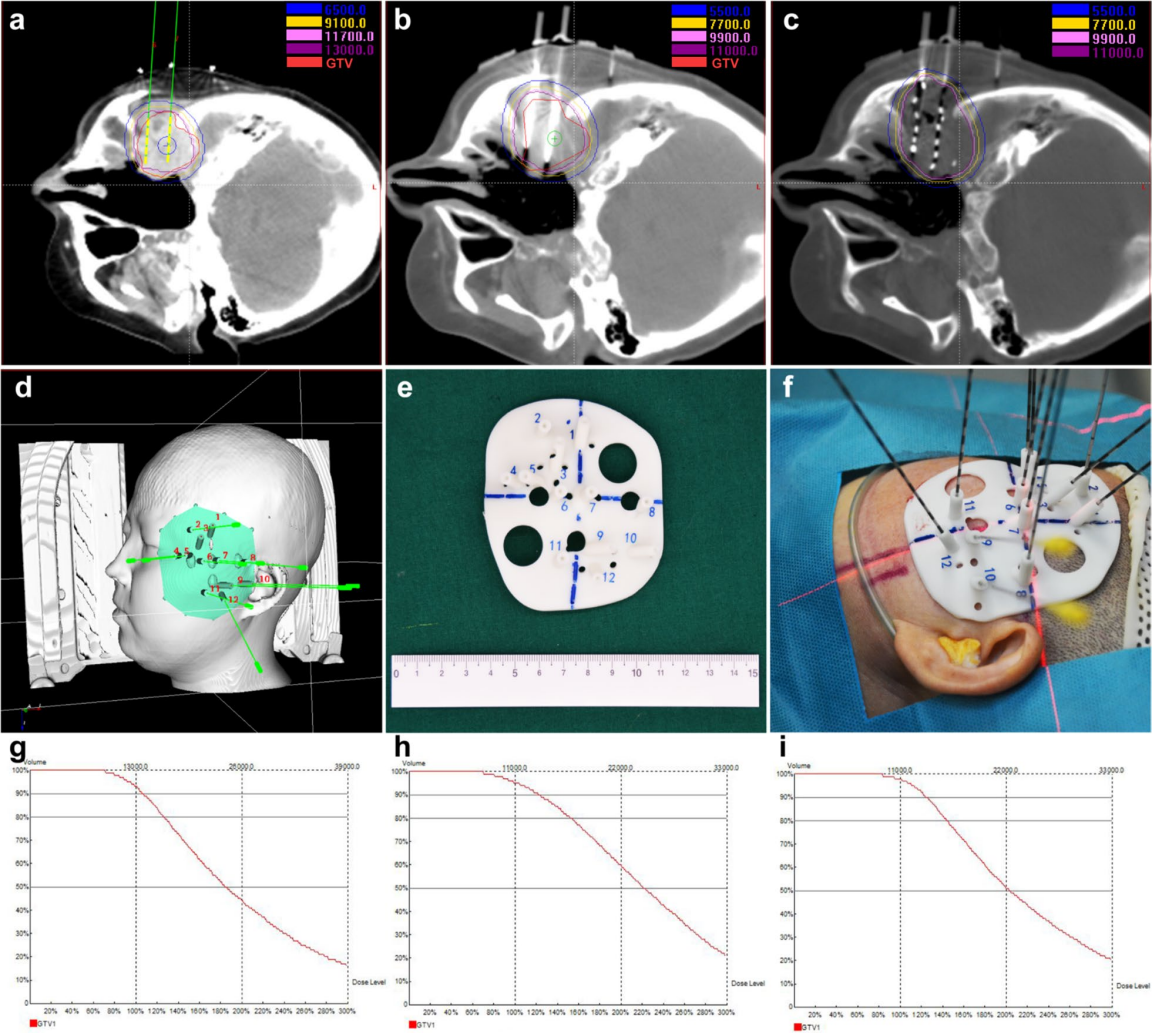
The CT-guided ^125^I seed implantation procedure and dose-volume histograms of gross tumor volume. Figure **a** showed the preoperative treatment plan including the planned needle locations, seed distribution, target volume doses, and organs at risk in a case of sinus osteosarcoma. The green needles and yellow seeds were the simulated needles and seeds in the brachytherapy treatment planning system. Figure **b** showed the actual locations of the needles before the implanting of seeds during operation. Figure **c** showed the actual distribution of seeds and the doses in target volume and organs at risk after seed implantation. Figure **d** showed the 3D-printed non-coplanar template model with guide holes on it in brachytherapy treatment planning system. Figure **e** showed the 3D-printed non-coplanar template model used in the implantation. Figure **f** showed the scene of seed implantation. Figure **g**–**i** showed the dose-volume histograms of gross tumor volume preoperation, intraoperation, and postoperation. The D90 before, in, and after ^125^I seed implantation were 138.6 Gy, 135.4 Gy, 137.4 Gy, respectively

In the treatment of HNC, it is important to maximize LC and minimize morbidity [34]. Permanent interstitial ^125^I seed implantation is one of the most promising brachytherapy techniques [35]. Permanent implantation of ^125^I seeds into tumors aims to provide high doses of radiation to the tumor, and the radiation outside the implanted volume falls very sharply, which can minimize the damage to the peripheral neurovascular structures and overlying skin [36]. Figure 1 showed the procedure of CT-guided ^125^I seed implantation and dose-volume histograms of gross tumor volume.

This technique possesses several advantages: (1) it is minimally invasive, and requires a short overall treatment duration, (2) dose distribution can be accurately predicted, (3) continuous radiation increases the possibility of destroying malignant cells during the cell cycle, (4) continuous LDR radiation from a low-energy source is effective against hypoxic components, which could be found in rapidly dividing cell populations, and (5) low incidence of acute adverse effects [37–39].

Additionally, LDR-BRT takes the advantage of the radiobiological properties of tumor cells (mainly redistributed in the cell cycle) and healthy tissues (DNA damage repair)[27]. Numerous studies have confirmed the efficacy and safety of LDR-BRT (Table 3) [34–41].

**Table 3:** Results of irradiation using LDR-BRT

Study	Study design	No. of patients	Previous radiotherapy	Follow-up, mo	Tumor volume, median (range) / Lesion size	Treatment technique	Median actuarial D90 (range), Gy	Efficacy	Toxicity	References
Yuliang Jiang, 2010	R	25	Median interval to failure from prior irradiation: NA; Median dose of prior irradiation: 70 Gy (range, 20–150 Gy)	8 (3–40)	NA	CT/ultrasound guided permanent ^125^I seed implantation	130 (90–160)	1-year LC: 48.7%; 2-year LC: 39.9%; Median local DFS: 12 mo; 1-year OS: 42.5%; 2-year OS: 28.3%; Median OS: 11 mo	None	[37]
Yuliang Jiang, 2010	R	14	Median time to previous irradiation: NA; Median dose of prior irradiation: 70 Gy (range, 50–250 Gy)	13 (3–60)	Tumor diameter < 7 cm	CT guided permanent ^125^I seed implantation	157.5 (90–218)	1-year LC: 52%; 2-year LC: 39%; 3-year LC: 39%; 5-year LC: 39%; Median LC: 18 mo; 1-year OS: 65%; 2-year OS: 39%; 3-year OS: 39%; 5-year OS: 39%; Median OS: 20 mo	Grade 1 skin reaction: 7.1%; Grade 1 mucosal reaction: 7.1%; Ulceration with tumor progression after 11 months: 7.1%	[38]
Ping Jiang, 2011	R	29	Median time to previous irradiation: NA; Median dose of prior irradiation: 70 Gy (range, 20–150 Gy)	8 (3–40)	NA	Ultrasound guided permanent ^125^I seed implantation	130 (90–160)	1-year LC: 53.1%; 2-year LC: 34.8%; 3-year LC: 17.4%; Median LC: 16 mo; 1-year OS: 54.1%; 2-year OS: 27.5%; 3-year OS: 27.5%; Median OS: 13 mo	None	[39]
Na Meng, 2012	R	17	Median time to previous irradiation: 10 mo (range, 1–80 mo); Median dose of prior irradiation: 70 Gy (range, 20–150 Gy)	10 (3–48)	Tumor diameter ≤ 5 cm	CT/ultrasound guided permanent ^125^I seed implantation	126 (90–160)	1-year LC: 66.5%; 2-year LC: 49.9%; Median LC: 16 mo; 1-year OS: 51.3%; 2-year OS: 38.5%; Median OS: 16 mo	One seed migration: 5.9%; One and two seeds loss: 11.8%; Temporary skin ulceration: 5.9%	[34]
Lihong Zhu, 2013	R	19	Median time to previous irradiation: NA; Median dose of prior irradiation: 64 Gy (range, 34–145 Gy)	11 (3–44)	Tumor diameter < 9 cm	CT/ultrasound guided permanent ^125^I seed implantation	131 (90–160)	1-year LC: 73.3%; 2-year LC: 27.5%; 3-year LC: 27.5%; Median LC: 24 mo; 1-year OS: 53.0%; 2-year OS: 18.2%; 3-year OS: 18.2% Median OS: 13 mo	Grade 1 skin reaction: 5.3%; Ulceration associated with tumor progression and died of local recurrence at 11 months after seed implantation: 5.3%	[36]
Ping Jiang, 2019	R	64	Median time to previous irradiation: NA; Median dose of prior irradiation: 60 Gy (range: 40–74 Gy)	14 (1–103.5)	Mean tumor volume: 8 (2.5–320) cm^3^	Ultrasound guided permanent ^125^I seed implantation	130 (90–160)	1-year LC: 75.2%; 3-year LC: 73.0%; 5-year LC: 69.1%; 1-year OS: 57.4%; 3-year OS: 31%; 5-year OS: 26.6%; Median OS: 20 mo	Grade 4 skin ulceration: 3.1%; Grade 1 or 2 skin reactions: 17.2%	[35]
Zhe Ji, 2019	R	101	Median time to previous irradiation: 18.8 mo (range, 1.3–213.8 mo); Median dose of prior irradiation: 66 Gy (range, 30–160 Gy)	12.2 (2.9–73.2)	Tumor volume: 15.5 (2.4–99.4) cm^3^	CT-guided ^125^I seed implantation	117 (44–246)	CR: 11.9%; PR: 48.5%; SD: 37.6%; PD: 2.0%; 1-year LC: 40.6%; 3-year LC: 26.6%; 5-year LC: 26.6%; Median LC: 10 mo; 1-year OS: 54.3%; 3-year OS: 15.5%; 5-year OS: 15.5%; Median OS: 15 mo	Grade 1 to 2 skin or mucosal toxicity: 15.8%; Grade 3 skin or mucosal toxicity: 7.9%; Grade 4 skin or mucosal toxicity: 2% Skin pain or mucosal reactions: 13.9%; Dry mouth aggravation: 2.0%	[40]
Yi Chen, 2020	R	25	Median time to previous irradiation: NA; Median dose of prior irradiation: 68 Gy (range, 50–115 Gy)	23 (6–82)	GTV: 60 (5–164) cm^3^	CT-guided ^125^I seed implantation	152 (106–179)	1-year LPFS: 65.6%; 3-year LPFS: 34.4%; 5-year LPFS: 22.9%; Median LPFS: 16.0 mo; 1-year OS: 70.8%; 3-year OS: 46.6%; 5-year OS:34.0%; Median OS: 28.0 mo	Severe pain during seed implantation: 8%; Hematoma in the neck: 4%; Grade 1 skin reaction: 12%; Grade 2 skin reaction: 8%; Grade 1 mucosal reaction: 8%; Grade 2 mucosal reaction: 4%; Grade 1 xerostomia:4%	[41]

*R* retrospective analysis, *mo* months, *NA* not available, *D90* the doses delivered to 90% of the target volume, *GTV* gross tumor volume, *LC* local control, *OS* overall survival, *LPFS* local progression-free survival

In 2010, Jiang et al. assessed the feasibility, efficacy, and morbidity of CT/ultrasonography-guided permanent percutaneous ^125^I seed implantation in the treatment of rHNSCC. They enrolled 25 patients who received CT/ultrasonography-guided permanent percutaneous ^125^I seed implantation, and the median actuarial D90 (the dose to 90% of the target volume) of the implanted ^125^I seeds was 130 Gy. The median follow-up was 8 months. The median local DFS was 12 months, and the 1- and 2-year LC rates were 48.7% and 39.9%, respectively. The median LC for nodal recurrence and primary recurrence was 12 and 16 months, respectively, with no significant difference. The 1- and 2-year survival rates were 42.5% and 28.3%, respectively. No patient had grade 4 or 5 toxicity [37].

A number of scholars studied 14 patients with rHNC who received CT-guided ^125^I seed implantation, and the post-plan showed that the median actuarial D90 of ^125^I seeds was 157.5 Gy. The median local control was 18 months, and the median survival was 20 months. Regarding complications, grade 1 skin reaction was found in 1 patient, 1 patient experienced grade 1 mucosal reaction, and 1 patient developed ulceration with tumor progression after 11 months. Among all patients, 28.6% (4/14), 7.1% (1/14), and 7.1% (1/14) of patients died of local recurrence, metastasis, and liver cirrhosis, respectively [38].

In 2011, Jiang et al. recruited 29 patients with rHNC who received ultrasonography-guided permanent percutaneous ^125^I seed implantation. The median actuarial D90 of ^125^I seeds was 130 Gy. The median local control was 16 months, while the median survival was 13 months. Among 25 patients, 5 and 7 patients died of local recurrence and metastasis, respectively. Besides, 2 patients had recurrence at 3 and 8 months after implantation, and subsequently died of pneumonia; 1 patient died of heart disease, and 1 patient developed ulceration as cancer progressed [39].

Additionally, a study evaluated 81 lesions of 64 patients, which were permanently implanted with ^125^I seeds under ultrasound guidance. According to the results, 27% and 53% of patients achieved CR and PR, respectively. The 1-, 3-, and 5-year tumor control rates were 75.2%, 73.0%, and 69.1%, respectively, while the median survival was 20 months. Severe complication was grade 4 skin ulceration in two patients with cervical lymph node recurrence who had previously received radiation therapy and had recurrent lesions invading the subcutaneous tissues prior to seeds implantation. Moreover, the 5-year LC rate of cervical lymph node recurrence was higher than that of the recurrence or residual lesions of primary HNC. D90 ≥ 130 Gy was noted as a positive prognostic factor for local tumor control, and location of recurrent lesions and time-to-progression (TTP, from the start of implantation to progression of the disease) were prognostic factors for survival. In addition, the advantages of ultrasound-guided seeds implantation include: (1) real-time guidance; (2) convenience and quick; (3) reproducible; (4) no additional radiation dose exposure, and disadvantages include: low image resolution and only 2D images can be obtained. Therefore, ultrasound-guided interstitial permanent ^125^I seeds implantation is a preferred option for patients with cervical lymph node recurrences or metastases from head and neck, superficial maxillofacial, and base of the tongue cancers, while tumor invasion into the skin is a contraindication [35].

In 2019, Ji et al. evaluated the efficacy and prognostic factors of CT-guided radioactive ^125^I seed implantation in the treatment of rHNC after EBRT. In total, 101 patients with rHNC underwent ^125^I seed implantation under CT guidance, and the median D90 was 117 Gy. The median LC was 10 months, and the median survival was 15 months. In addition, nonsquamous cell carcinoma, D90 ≥ 120 Gy, lesion volume ≤ 20 cm^3^, and short-term efficacy (CR + PR) were correlated with better LC. High Karnofsky performance status (KPS) and lesion volume ≤ 20 cm^3^ were independent factors associated with survival [40].

**Fig. 2:**
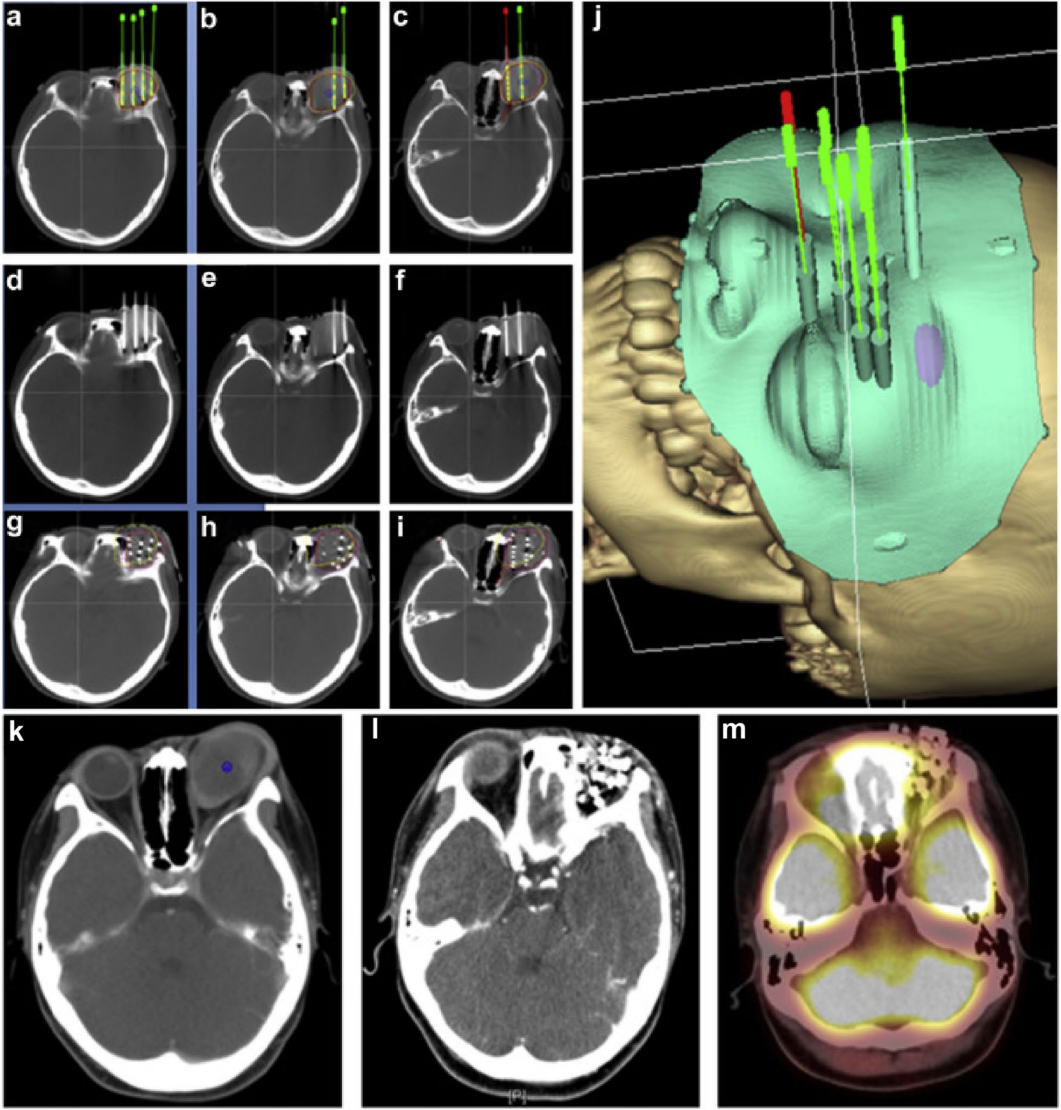
The CT-guided ^125^I seed implantation procedure and therapeutic efficacy. Figure **a**–**c** showed the preoperative treatment plan, including the planned needle locations, seed distribution, target volume doses, and organs at risk in a case of locally recurrent embryonal rhabdomyosarcoma of the orbit after surgery and EBRT. The green or red needles and yellow seeds were the simulated needles and seeds in the brachytherapy treatment planning system. Figure **d**–**f** showed the actual locations of the needles before the seed implantation. Figure **g**–**i** showed the actual distribution of seeds and the doses in target volume and organs at risk after seed implantation. Figure **j** showed the 3D-printed non-coplanar template model with guide holes on it in brachytherapy treatment planning system. Figure **k**–**m** showed CT or PET-CT images of the tumor in preoperation, 3-month postoperation, and 6-month postoperation, and PET-CT showed no residual tumor with metabolic activity in Figure **m** [41]

Chen et al. evaluated the efficacy and safety of radioactive ^125^I seed implantation under the guidance of CT as a salvage treatment for 25 patients with locally recurrent head and neck soft tissue sarcoma (rHNSTS) after surgery and EBRT. The median D90 was 152 (range, 106–179) Gy. When ^125^I seeds were implanted, the objective response rate (ORR) was 76.0%. The 1-, 3-, and 5-year LPFS rates were 65.6%, 34.4%, and 22.9%, respectively, and the median LPFS was 16.0 months. The 1-, 3-, and 5-year OS rates were 70.8%, 46.6%, and 34.0%, respectively, and the median OS was 28.0 months. In addition, recurrent T stage and histological grade were found as prognostic factors for LPFS, while histological grade was noted as a predictor of OS [41]. The procedure and therapeutic efficacy of CT-guided ^125^I seed implantation in a case of locally recurrent embryonal rhabdomyosarcoma were shown in Fig. 2.

Permanent interstitial ^125^I seed implantation can provide targeted radiation to ensure that tumor cells are continuously killed for several months, and it also avoids high morbidity associated with EBRT or surgery. In recent studies, the 1-, 2-, 3-, 5-year LC ranged from 40.6% to 75.2%, 27.5% to 49.9%, 17.4% to 73.0%, 26.6% to 69.1%, and the 1-, 2-, 3-, 5-year OS ranged from 42.5% to 70.8%, 18.2% to 39%, 15.5% to 46.6%, 15.5% to 39%. The median LC was 10–24 months, and the median OS was 11–28 months. D90, tumor histological type, lesion volume, and short-term efficacy were prognostic factors for LC, and location of recurrent lesions, TTP, KPS, lesion volume and histological grade were associated with OS. Through LDR-BRT treatment, patients can achieve a better LC, and a lower incidence of toxicity can be attained.

## Irradiation with systemic therapy

The pioneering studies of the Radiation Therapy Oncology Group (RTOG) 9610 and 9911 have shown that conventional hypofractionated re-irradiation plus systemic therapy could achieve a 2-year survival rate of 15–26% in rHNSCC patients. However, the conventional re-irradiation resulted in severe (grade ≥ 3) acute and late toxicities with incidence rates of 63–78% and 22–37%, respectively. The median OS in these trials was only slightly higher than that of chemotherapy alone [22]. With the development of radiotherapy, a great number of scholars have investigated the therapeutic effects of the combination of radiotherapy and systemic therapy on the treatment of patients with rHNC (Table 4)[42–53].

**Table 4:** Results of irradiation with systemic therapy

Study	Study design	No. of patients	Previous radiotherapy	Irradiation technology	Medicine	Follow-up, mo	Tumor volume, median (range), cm^3^	No. of fractions, median (range)	Total dose, median (range), Gy	Efficacy	Toxicity	References
Felix Zwicker, 2011	R	10	Median time to previous irradiation: 52 mo; Median dose of prior irradiation: 66 Gy (range, 30–71.4 Gy)	IMRT	Cetuximab	6.5 (0.5–16)	PTV: 182.5 (48–1320)	28 (22–36)	50.4 (39.6–64.8)	1-year LC: 61%; 1-year LRC: 44%; 1-year DMFS: 75%; 1-year OS: 40%; Median OS: 7 mo	Severe acute toxicity: Fatal infield arterial bleeding: 10%; Flap necrosis: 10%; Severe late toxicities: Fibrosis of the temporomandibular joint: 10%; Stenosis of the cervical esophagus: 10%	[45]
Jacques Tortochaux, 2011	Randomized phase III trial	30	Time to previous irradiation ≥ 6 mo; Median dose of prior irradiation: 62.5 Gy	Conventional treatment planning system or 3D-CRT	Fluorouracil and hydroxyurea	NA (maximum: 60)	NA	30	60	6-month OS: 50%; 9-month OS: 30%; 1-year OS: 23%; Median OS: 6 mo	Grade ≥ 3 acute toxicities: Mucositis:6.7%; Radio-dermatitis: 3.3%; Thrombocytopenia: 6.7%; Leukopenia: 10%; Anemia: 3.3%; Stomatitis and pulmonary adverse event: 10%; Infectious shock: 3.3% Grade ≥ 3 late toxicities: 36.7%	[42]
Dwight E Heron, 2011	Retrospective matched cohort study	35	Median time to previous irradiation: 18.4 mo (range, 8.3–311.5 mo); Median dose of prior irradiation: 69.2 Gy (range, 36–129.5 Gy)	SBRT	Cetuximab	15.9 (3.1–36.5)	28.7 (5–91.7)	5	40 (20–44)	1-year LC: 78.6%; 2-year LC: 49.2%; Median LC: 23.8 mo; 1-year OS: 66%; 2-year OS: 53.3%; Median OS: 24.5 mo	Grade 3 acute toxicities: Xerostomia: 2.9%; Dysphagia: 2.9%; Grade 3 late toxicities: Xerostomia: 2.9%; Dysphagia: 2.9%	[50]
		35	Median time to previous irradiation: 19.2 mo (range, 4.4–269.7 mo); Median dose of prior irradiation: 68 Gy (range, 32–140 Gy)	SBRT (matched)	None	14.6 (2.2–39)	29.0 (4.8–86.8)	5	40 (20–44)	1-year LC: 53.8%; 2-year LC: 33.6%; Median LC: 13.2 mo; 1-year OS: 52.7%; 2-year OS: 21.1%; Median OS: 14.8 mo	Grade 3 acute toxicities: Xerostomia: 2.9%; Grade 3 late toxicities: Xerostomia: 2.9%	
L Vormittag, 2012	Prospective phase II trial	31	Median interval to failure from prior irradiation: 15 mo; Median dose of prior irradiation: 62 Gy (range, 45–80 Gy)	3D treatment planning radiotherapy	Capecitabine	NA	NA	25 (15–30)	50 (30–60)	CR: 19%; PR: 48%; 1-year OS: 42%; 2-year OS: 10%; Median OS: 8.4 mo	Grade 3 or 4 mucositis: 16.1%; Grade 3 skin reactions: 6.5%; Grade 3 anemia: 3.2%; Grade 3 late toxicities: Xerostomia: 7.7%; Dysphagia: 23%; Pain: 7.7%	[43]
Jordan Kharofa, 2012	R	38	Median time to previous irradiation: 28 mo (range, 3–228 mo); Median dose of prior irradiation: 68 Gy (54–70 Gy)	IMRT: 76%; 3D-CRT: 24%	Carboplatin and paclitaxel	16	NA	30	60	1-year PFS: 44%; 2-year PFS: 34%; 5-year PFS: 29%; Median TTP: 7 mo; 1-year OS: 54%; 3-year OS: 31%; 5-year OS: 20%; Median OS: 16 mo	Acute toxicities: Grade 2 neutropenia: 5%; Grade 3 neutropenia: 15%; Grade 1/2 thrombocytopenia: 8%; Significant late toxicities: 16%	[44]
Eric F Lartigau, 2013	Prospective phase II trial	60	Mean time to previous irradiation: 38 mo; Median dose of prior irradiation: NA	SBRT	Cetuximab	11.4	Median tumor size: 29 mm	6	36	Median PFS: 7.1 mo; 1-year OS: 47.5%; Median OS: 11.8 mo	Toxic death from hemorrhage and denutrition: 1.8%; Grade 3 toxicities: 32.1%	[51]
D Milanović, 2013	R	23	Median time to previous irradiation: 102.2 mo (range, 12–293 mo); Median dose of prior irradiation: 64.3 Gy (range, 50–70 Gy)	Re-irradiation	Cetuximab	11.04 (1–37)	NA	NA (28–36)	NA (50.4–66)	1-year OS: 34.8%; Median OS: 9 mo; Median PFS: 4.3 mo	Grade 3 acute toxicities: Dermatitis: 35%; Dysphagia: 30%; Acneiform rash: 30%; Mucositis:15%; Voice change: 15%; Pain: 9.6%; Grade 3 late toxicities: Dysphagia: 17.6%; Pain: 17.6%; Fibrosis: 11.8%; Voice changes: 5.9%; Xerostomy: 5.9%; Trismus: 5.9%	[46]
John A Vargo, 2015	Prospective phase II trial	48	Median time to previous irradiation: 18 mo (range, 3–423 mo); Median dose of prior irradiation: 70 Gy (52.5–118.2 Gy)	SBRT	Cetuximab	18 (10–70)	GTV: 36.5 (3.6–209.2)	5	NA (40–44)	1-year LPFS: 60%; 1-year locoregional PFS: 37%; 1-year distant PFS: 71%; 1-year PFS: 33%; 1-year OS: 40%; median OS: 10 mo	Acute grade 3 toxicities: 6%; Late grade 3 toxicities: 6%	[52]
Nicolas Dornoff, 2015	Retrospective comparative study	33	Median time to previous irradiation: 24 mo (range, 5–281 mo); Median dose of prior irradiation: 64 Gy	3D-CT-based conformal radiotherapy	Cetuximab	18.3	GTV: 35 (2.9–213); PTV: 159.5 (22.8–1345)	28	50.4 (1.8–72)	1-year OS: 44.4%; 2-year OS: 11.9%; 1-year LC: 46.4%; 1-year FFM: 73.6%	Grade ≥ 3 acute toxicities: 57.6%; Grade ≥ 3 late toxicities: Dysphagia: 21.2%; Trismus: 6.1%; Xerostomia: 6.1%	[48]
		33		3D-CT-based conformal radiotherapy	Cisplatin with/without 5-fluorouracil					1-year OS: 45.5%; 2-year OS: 30.3%; 1-year LC: 54.2%; 1-year FFM: 81%	Grade ≥ 3 acute toxicities: 51.5%; Grade ≥ 3 late toxicities: Lymphedema: 3.1%; Dysphagia: 21.2%	
M Ritter, 2016	R	18	Median interval to failure from prior irradiation: 2.3 mo (range, 2–8 mo); Median dose of prior irradiation: 68.1 Gy (range, 50–105 Gy)	HDR-BRT	Cetuximab plus paclitaxel	13.4 (0.1–72.9)	CTV (V100): 61.9 (22.3–149.5)	10.6 (6–14)	27.0 (15–35)	1-year DFS: 32%; 2-year DFS: 24%; 1-year OS: 85%; 2-year OS: 65%	Grade 3 acute toxicities: 11.1%; Grade 3 late toxicities: 5.6%	[53]
		18	Median interval to failure from prior irradiation: 38.1 mo (range, 18–99 mo); Median dose of prior irradiation: 66.2 Gy (range, 59–77 Gy)	HDR-BRT (matched)	None		CTV (V100): 46.4 (4.0–111.1)	10.8 (8–12)	27.1 (20–30)	1-year DFS: 17%; 2-year DFS: 17%; 1-year OS: 68%; 2-year OS: 58%	Grade 3 acute toxicities: 11.1%; Grade 3 late toxicities: 5.6%	
M J Awan, 2018	Prospective phase II trial	45	Median time to previous irradiation: 30 mo (range, 6–219.6 mo); Median dose of prior irradiation: 70 Gy (range, 63–75.6 Gy)	IMRT	Cisplatin plus cetuximab	16.6	NA	30	60 (60- 70.29)	1-year RFS: 34.1%; 2-year RFS: 27.3%; 1-year OS: 60.4%; 2-year OS: 45.3%	Grade ≥ 3 acute toxicities: Lymphopenia: 46%; Pain: 22%; Dysphagia: 13%; Radiation dermatitis: 13%; Mucositis: 11%; Anorexia: 11%; Grade 3 late toxicities: 17.4%	[47]
Yungan Tao, 2018	RCT	26	Median time to previous irradiation: NA; Dose of prior irradiation ≥ 50 Gy	3D-CRT with or without intensity-modulation (60 Gy over 11 weeks radiation, six cycles with each cycle delivering 2 Gy/fraction, 5 days/week)	5FU and hydroxyurea	36	NA	30	60 (60–80)	Median DFS: 11.2 mo; 2-year OS: 45%; Median OS: 21.9 mo	Grade 3 or 4 toxicities: End of reirradiation: 42.3%; 6 months from randomization: 28.0%; 12 months from randomization: 17.6%; 24 months from randomization: 0%	[49]
		27		3D-CRT with or without intensity-modulation (60 Gy over 5 weeks radiation, 1.2 Gy twice daily)	Cetuximab	36	NA	50 (bid)	60	Median DFS: 12.0 mo; 2-year OS: 67%; Median OS: 37.4 mo	Grade 3 or 4 toxicities: End of reirradiation: 37.0%; 6 months from randomization: 20.0%; 12 months from randomization: 27.8%; 24 months from randomization: 14.3%	

*R* retrospective analysis, *RCT* randomized controlled trial, *mo* months, *NA* not available, *IMRT* intensity-modulated radiation therapy, *3D-CRT* three-dimensional conformal radiotherapy, *SBRT* stereotactic body radiation therapy, *HDR-BRT* high-dose-rate brachytherapy, *PTV* planning target volume, *CTV* clinical target volume, *GTV* gross tumor volume, *LC* local control, *LRC* locoregional control, *DMFS* distant metastasis-free survival, *OS* overall survival, *CR* complete response, *PR* partial response, *PFS* progression-free survival, *TTP* time-to-progression, *LPFS* local progression-free survival, *FFM* freedom from metastases, *RFS* recurrence-free survival, *DFS* disease free survival

### Studies reporting the application of 3D-CRT or IMRT combined with systemic therapy

Numerous studies have recently reported the combination of radiotherapy with systemic chemotherapy for rHNC. Tortochaux et al. assessed the effects of methotrexate versus concurrent re-irradiation, fluorouracil, and hydroxyurea on patients who received palliative treatment for recurrent or second primary HNSCC (primary tumor site without nasopharynx). A total of 57 patients with recurrent or second primary HNSCC were randomized to the concurrent re-irradiation (using conventional treatment planning system or 3D-CRT), fluorouracil, and hydroxyurea arm (R-RT arm, 30 patients) or the methotrexate arm (Ch-T arm, 27 patients). In the R-RT arm, the median dose of irradiation was 60 Gy. All patients died within the longest follow-up of 5 years. The R-RT arm achieved 4 CRs, while no CR was achieved in the Ch-T arm. However, re-irradiation did not improve OS compared with methotrexate alone (23% vs. 22% at 1-year). Compared with previously reported studies, the survival rate with re-irradiation and chemotherapy was poor in this study, possibly because patients were selected for palliative therapy. Moreover, 11 patients had grade ≥ 3 late toxicities in the R-RT group, and 5 patients in the Ch-T group. The results confirmed that in patients with recurrent or second primary HNSCC who received palliative treatment, concurrent re-irradiation, fluorouracil, and hydroxyurea, no improvement in OS was detected compared with the administration of methotrexate alone [42].

Vormittag et al. investigated the safety and efficacy of 3D treatment planning radiotherapy combined with capecitabine in 31 patients who had rHNSCC (primary tumor site without nasopharynx) within a previously irradiated field. The median dose was 50 Gy. The ORR was 68%, including 6 (19%) patients who achieved CR. The median OS was 8.4 months, and grade 3 or 4 mucositis occurred in 4 patients and 1 patient, respectively. Skin reactions of grade 3 were observed in 2 (6%) patients. Besides, 1 patient (3%) had grade 3 anemia [43].

Kharofa et al. evaluated the efficacy and toxicity of a continuous course, conformal re-irradiation with paclitaxel and carboplatin in the treatment of locally recurrent, non-metastatic HNSCC in the previous irradiated field. A total of 38 non-metastatic rHNSCC patients received re-irradiation (IMRT: 76%; 3D-CRT: 24%) at a median dose of 60 Gy. The median TTP was 7 months, and the 1-, 2-, and 5-year PFS rates were 44%, 34%, and 29%, respectively. The median OS was 16 months, and the 1-, 3-, and 5-year survival rates were 54%, 31%, and 20%, respectively. Severe acute toxicity was found as grade 3 neutropenia (15%), while significant late toxicities were experienced in 6 (16%) patients following completion of re-irradiation [44].

Several scholars have concentrated on the use of novel EGFR-targeted drugs (e.g., cetuximab) as radiosensitizers to further improve disease outcomes without increasing toxicity [54]. Milanović et al. studied 23 previously irradiated, inoperable recurrent or second primary HNSCC (primary tumor site without nasopharynx) patients. Cetuximab was used as a loading dose 2 days before radiotherapy (400 mg/m^2^), followed by a weekly concurrent dose (250 mg/m^2^). One patient died of anaphylactic shock during the first administration of cetuximab and 2 patients were excluded according to their requests. In total, 20 patients completed re-irradiation (50.4–66.6 Gy) and received cetuximab as prescribed. In addition, the 1-year survival rate was 34.8%, and the median OS and PFS were 9 and 4.3 months, respectively. Grade 3 acute toxicities were dermatitis (35%), dysphagia (30%), acneiform rash (30%), mucositis (15%), voice change (15%), and pain (9.6%). Grade 3 late toxicities were dysphagia (17.6%), pain (17.6%), fibrosis (11.8%), voice changes (5.9%), xerostomia (5.9%), and trismus (5.9%). Multivariate regression analysis showed that acneiform rashes had a significant positive effect. Besides, if the interval from the first radiotherapy to re-irradiation was more than 120 months, survival was significantly shorter, and the authors speculated that these patients had a more aggressive second primary/radiation-induced cancer [46].

Awan et al. enrolled 45 rHNSCC patients who completed the treatment. Patients with squamous cell carcinoma of salivary gland or nasopharynx were excluded from this study. Among them, 33 patients had undergone surgical resection before re-irradiation. Cetuximab (400 mg/m^2^) was given as a loading dose in the first week, followed by cetuximab (250 mg/m^2^) and cisplatin (30 mg/m^2^) that were administered weekly concurrent with IMRT at a dose of 60–66 Gy for 6 consecutive weeks. The median follow-up was 1.38 years. Moreover, the 1-year OS was 60.4%, and the 1-year recurrence-free survival was 34.1%. There was no grade 5 acute toxicity. Besides, 8 patients experienced grade 3 late toxicities, in which swallowing was found in 4 patients. Importantly, young age played a positive role in improving OS. OS was not associated with radiation dose, surgery before re-irradiation, or the interval from previous EBRT [47].

Furthermore, some studies have compared the use of radiotherapy with different systemic therapies. Dornoff et al. evaluated the efficacy and toxicity of cisplatin or cetuximab combined with re-irradiation therapy (3D-CT-based conformal radiotherapy) in the treatment of patients with unresectable rHNSCC. A total of 66 patients with rHNSCC, with previously irradiated areas, received re-irradiation with either cetuximab (n = 33) or cisplatin-based chemotherapy (n = 33). The median re-irradiation dose for all patients was 50.4 Gy. With a mean follow-up of 18.3 months, the 1-year survival rate of the cetuximab arm and cisplatin arm was 44.4% and 45.5%, respectively. At 1 year, the LC rates were 46.4% and 54.2% (P = 0.625), and the freedom from metastasis rates were 73.6% and 81% in the two arms, respectively. Hematological toxicity of grade ≥ 3 in the cisplatin arm was more frequent, while the pain of grade ≥ 3 in the cetuximab arm was noteworthy. Additionally, hemoglobin levels and the interval between primary radiotherapy and re-irradiation were found as important prognostic factors for OS. Positive effects of the longer radiotherapy and re-irradiation interval on the prognosis were reported, which could reflect the low biological invasiveness or the second primary lesion in the irradiated area. The hemoglobin level before treatment was a predictor of survival for patients with primary HNSCC undergoing radiotherapy, and re-irradiation was confirmed as well. Anemia indicated the presence of comorbidities and organ dysfunction, and hypoxia was associated with the mechanism of radioresistance [48].

Tao et al. compared two methods of re-irradiation in terms of survival and toxicity. There were 26 patients with recurrent or second primary HNSCC, with the previously irradiated area, who were randomly assigned to receive 3D-CRT with or without intensity-modulation for a dose of 60 Gy over 11 weeks plus 5FU-hydroxyurea (Vokes’ protocol, VP-arm), while 27 patients received 60 Gy (1.2 Gy twice daily) over 5 weeks plus cetuximab (hypofractionated radiotherapy, HFR-arm). The results showed that there was no significant difference in OS (median OS: 37.4 vs. 21.9 months, P = 0.12), toxicity, and DFS between HFR-arm and VP-arm [49].

Thus, patients with rHNC may profit from systemic therapy with 3D-CRT/IMRT. In recent studies, the 1-, 2-year OS ranged from 23% to 60.4%, 10% to 67%, and the median OS was 6–37.4 months. However, the efficacy and safety of these therapeutic regimens remain to be further verified.

### Studies reporting the application of SBRT with systemic therapy

Heron et al. compared SBRT alone and SBRT with weekly cetuximab infusion in the management of locally rHNSCC. The median dose in both groups was 40 Gy. The results indicated that compared with SBRT alone, concurrent cetuximab with SBRT had an advantage in OS without a significant increase in the incidence of grade 3/4 toxicities. This survival-based advantage was also observed in patients that received cetuximab in the previous treatment regimen. In addition, SBRT dose, nasopharynx primary site, and KPS score predicted for better OS [50].

Lartigau et al. enrolled 60 patients with inoperable recurrent, or new primary tumors in previously irradiated areas, who were treated with SBRT and 5 injections of cetuximab. Patients also received a trial dose of 400 mg/m^2^ cetuximab 1 week before SBRT. The re-irradiation dose was 36 Gy in 6 fractions. During the 2 weeks of SBRT and the following 2 weeks, patients received 4 injections of cetuximab every week at a dose of 250 mg/m^2^. The median follow-up was 11.4 months, and the 1-year survival rate was 47.5%. At 3 months, the ORR was 58.4%, and the disease control rate was 91.7%. The median PFS was 7.1 months. Cutaneous toxicity was found in 41 patients, and one patient died of hemorrhage and malnutrition [51].

Vargo et al. studied 48 patients with locoregionally inoperable rHNSCC (primary tumor site without nasopharynx) who were treated with SBRT plus cetuximab. Patients with tumor volume < 25 cm^3^ received 40 Gy, while those with tumor volume ≥ 25 cm^3^ received 44 Gy. The 1-year LPFS was 60%, the locoregional PFS was 37%, the distant PFS was 71%, and the PFS was 33%. The median OS was 10 months, and the 1-year survival rate was 40%. Acute and late grade 3 toxicities were detected in 6% of patients respectively. Moreover, recurrent GTV (< 25 cm^3^) was associated with improved OS (1 year, 70%, and 22%) and locoregional PFS (1 year, 53%, and 22%) [52].

Therefore, the 1-year OS ranged from 40% to 66%, and the median OS was 10–24.5 months in recent studies using SBRT plus cetuximab regimen. As SBRT has a hypofractionation scheme, rHNC patients may profit from the combination of systemic therapy and SBRT within a shorter treatment time, which is highly appropriate for patients with a poor prognosis.

### Studies reporting the application of HDR-BRT with systemic therapy

Ritter et al. reported a second-line treatment, that is function-preserving surgical debulking, and then combined with postoperative interstitial brachytherapy and a simultaneous regimen of cetuximab and paclitaxel. The study group included 18 patients who had developed progressive disease after the first- or second-line therapy within a short time. Palliative treatment was given to patients with the advanced locoregional disease who failed to respond to (radio) chemotherapy. The mean total dose of HDR-BRT was 27.0 (range, 15–35) Gy, and 94% of patients were treated with surgical resection. The average DFS and OS in the study group were 8.7 and 14.8 months, respectively. In the control group (only including function-preserving surgical debulking and brachytherapy), the DFS and OS were 3.9 and 6.1 months, respectively. This indicated a positive trend by the additional use of the cetuximab plus taxane regimen. However, the validity of this study was limited due to the small number of patients [53].

Therefore, re-irradiation via HDR-BRT combined with concurrent cetuximab is a feasible regimen, which is accompanied by a low incidence of toxicity for rHNC patients.

## Conclusions

With the development of radiotherapy technology, its effects on patients with rHNC have been further improved. In particular, for patients who cannot undergo surgery, radiotherapy for recurrence is associated with a better prognosis. After salvage surgery or inoperable recurrence, patients can achieve long-term survival via radiotherapy. As the novel technologies of irradiation, IMRT, SBRT, HDR-BRT, and LDR-BRT have shown different characteristics. The comparison of different radiotherapy techniques for the irradiation of rHNC was shown in Table 5. Oncologists should pay further attention to the important role of radiotherapy in the treatment of rHNC. Besides, additional clinical research on the application of radiotherapy for diverse types of cancer is required for more reliable medical evidence, so that more patients can receive effective radiotherapy programs.

**Table 5:** Comparison of different radiotherapy techniques for irradiation of rHNC

Radiotherapy technique	Most frequent subsites treated	Median irradiation dose, Gy	Efficacy	Severe toxicity
IMRT	Oropharyngeal; Neck	49–70	2-year LC: 46–57%; 2-year OS: 41–50%	Grade ≥ 3 late toxicities: 14.2–57.1%; Grade 5 late toxicities: 1.3–7.4%
SBRT	Neck; Oral cavity	30–48	2-year LRC: 31.7–64%; 1-year OS: 32–58.9%; 2-year OS: 16–35%; Median OS: 7.5–14.4 mo	Grade ≥ 3 toxicities: 4.7–21%
HDR-BRT	Neck; Oral cavity;	12–36.5	1-year LC: 55–77%; 2-year LC: 62–67%; 1-year OS: 63–77%; 2-year OS: 42–67%; Median OS: 8.5–33.4 mo	Grade ≥ 3 toxicities: 0–34.4%
LDR-BRT	Neck; Nasopharynx	D90: 117–157.5	1-year LC: 40.6–75.2%; 2-year LC: 27.5- 49.9%; 1-year OS: 42.5–70.8%; 2-year OS: 18.2–39%; Median LC: 10–24 mo; Median OS: 11–28 mo	Grade ≥ 3 toxicities: 0–9.9%
Irradiation with systemic therapy	Oropharynx; Oral cavity	27–60	1-year LC: 46.4–78.6%; 1-year OS: 23–85%; 2-year OS: 10–67%; Median OS: 6–37.4 mo	Grade ≥ 3 toxicities: 5.8–57.6%

*mo* months, *IMRT* intensity-modulated radiation therapy, *SBRT* stereotactic body radiation therapy, *HDR-BRT* high-dose-rate brachytherapy, *LDR-BRT* low-dose-rate brachytherapy, *LC* local control, *LRC* locoregional control, *OS* overall survival

## Data Availability

Not applicable.

## Abbreviations

HNC: Head and neck cancer
rHNC: Recurrent head and neck cancer
HNSCC: Head and neck squamous cell carcinoma
rHNSCC: Recurrent head and neck squamous cell carcinoma
HPV: Human papillomavirus
IMRT: Intensity-modulated radiation therapy
SBRT: Stereotactic body radiation therapy
HDR-BRT: High-dose-rate brachytherapy
LDR-BRT: Low-dose-rate brachytherapy
CTCAE: Common Terminology Criteria for Adverse Events
RTOG: Radiation Therapy Oncology Group
EORTC: European Organization for Research and Treatment of Cancer
RSPHNC: Recurrent or second primary head and neck cancer
LC: Local control
OS: Overall survival
CI: Confidence interval
RT: Radiotherapy
OARs: Organs at risk
EBRT: External beam radiotherapy
LRC: Locoregional control
GTV: Gross tumor volume
CR: Complete response
PR: Partial response
SD: Stable disease
PFS: Progression-free survival
PTV: Planning target volume
CBOS: Carotid blow-out syndrome
RPA: Recursive partitioning analysis
Pt: Platinum
CT: Computed tomography
DFS: Disease-free survival
3D-CRT: Three-dimensional conformal radiotherapy
IORT: Intraoperative radiotherapy
LPFS: Local progression-free survival
TTP: Time-to-progression
KPS: Karnofsky performance status
rHNSTS: Recurrent head and neck soft tissue sarcoma
ORR: Objective response rate
